# Design of a Sensitive Balloon Sensor for Safe Human–Robot Interaction

**DOI:** 10.3390/s21062163

**Published:** 2021-03-19

**Authors:** Dongjin Kim, Seungyong Han, Taewi Kim, Changhwan Kim, Doohoe Lee, Daeshik Kang, Je-Sung Koh

**Affiliations:** Department of Mechanical Engineering, Ajou University, San 5, Woncheon-dong, Yeongtong-gu, Suwon 443-749, Korea; rlaehdwlswt@ajou.ac.kr (D.K.); sy84han@ajou.ac.kr (S.H.); rlaxodnl@ajou.ac.kr (T.K.); kchwan108@ajou.ac.kr (C.K.); sktd4@ajou.ac.kr (D.L.)

**Keywords:** pressure sensor, human–robot interaction, soft robot

## Abstract

As the safety of a human body is the main priority while interacting with robots, the field of tactile sensors has expanded for acquiring tactile information and ensuring safe human–robot interaction (HRI). Existing lightweight and thin tactile sensors exhibit high performance in detecting their surroundings. However, unexpected collisions caused by malfunctions or sudden external collisions can still cause injuries to rigid robots with thin tactile sensors. In this study, we present a sensitive balloon sensor for contact sensing and alleviating physical collisions over a large area of rigid robots. The balloon sensor is a pressure sensor composed of an inflatable body of low-density polyethylene (LDPE), and a highly sensitive and flexible strain sensor laminated onto it. The mechanical crack-based strain sensor with high sensitivity enables the detection of extremely small changes in the strain of the balloon. Adjusting the geometric parameters of the balloon allows for a large and easily customizable sensing area. The weight of the balloon sensor was approximately 2 g. The sensor is employed with a servo motor and detects a finger or a sheet of rolled paper gently touching it, without being damaged.

## 1. Introduction

There have been a number of attempts to push the technological boundaries of safety systems for safe human–robot interaction (HRI) [1,2,3,4]. A control strategy for robot manipulators, development of soft robotics, and sensing techniques to understand its surroundings not only provide the desired operation but also quantitative safety for humans. Protection of the human body is necessary to be considered prior to performing tasks. Robots must always be ready to reduce or avoid undesired impacts that may cause injuries. Touch sensing is a promising way to provide information to perform tasks and avoid potential dangers [5,6,7,8].

Tactile sensors can be categorized into three types with respect to their sensing areas: single-point contact sensors, high-spatial-resolution tactile arrays, and large-area tactile sensors [8]. The single-point contact sensor [9] and high spatial resolution tactile array [10,11] are able to sense external contact with high sensitivity and acquire the position of the contact force. However, the limitation of its sensing area generally demands more expensive and complex fabrication and data acquisition systems than large-area tactile sensors to cover the entire area of bulky and rigid robots, where high spatial resolution is not essential. For conventional robots, such as industrial robotic arms, which are potentially dangerous on a human scale, it is efficient to employ large-area tactile sensors to prevent any possible damage.

To cover a large area of the curved surface of the robots, the large-area tactile sensor must be flexible and light enough to be attached to robots; the sensors with a thin form factor have these characteristics [12,13,14,15,16]. These light and thin sensors minimize the interruption of the operation while being attached to acquire the necessary information. However, accidental collisions can still occur. The malfunction of rigid robots and sudden external impact by humans to which an actuator cannot react can be dangerous even with highly sensitive and thin tactile sensors.

Integrating rigid robots with inflatable structures and sensors can effectively alleviate such unexpected collisions while sensing the surrounding environments. An inflatable sleeve integrated with capacitive sensors or potentiometers acquires tactile information and reduces the impact force by collision for legged robots [17] or lightweight soft robotic arms [18]. Air-filled force-sensing modules at various scales provide contact force feedback and absorb impact [19,20,21]. In addition to integrating the inflatable structure with rigid robots, almost entirely inflatable robots [22,23,24,25] have favorable properties such as light weight, high flexibility, and shock-absorbing ability.

A majority of soft robots, especially those with inflatable structures, are actuated and controlled based on data from air pressure sensors. There are many commercialized pressure sensors that are sufficiently small and light to be employed in soft robots. However, to acquire data with high resolution and accuracy, the pressure sensor needs to be bulky to implement an integrated circuit amplifier. Conventional air pressure sensors also require flexible tubing connected to an inflatable body that allows air or fluid to flow through it. The limitations of conventional pressure sensors can present a challenge in designing and actuating untethered, small, and lightweight soft robots.

Herein, we present a lightweight and large-area sensing balloon sensor integrated with a highly sensitive strain sensor that detects any small contact force on its entire membrane and absorbs external impact. We used a mechanical crack-based sensor [26,27] that is highly sensitive to a strain with a gauge factor of over 2000 and as flexible as a 10-µm polyurethane acryl (PUA) film. This resistivity-based flexible sensor can be fabricated using different kinds of polymer films such as LDPE, polyimide (PI), and polyethylene terephthalate (PET) [28,29]. Crack-based strain sensors with polymer-type substrates can be easily integrated with a thermoplastic sheet-type inflatable structure.

**Figure 1:**
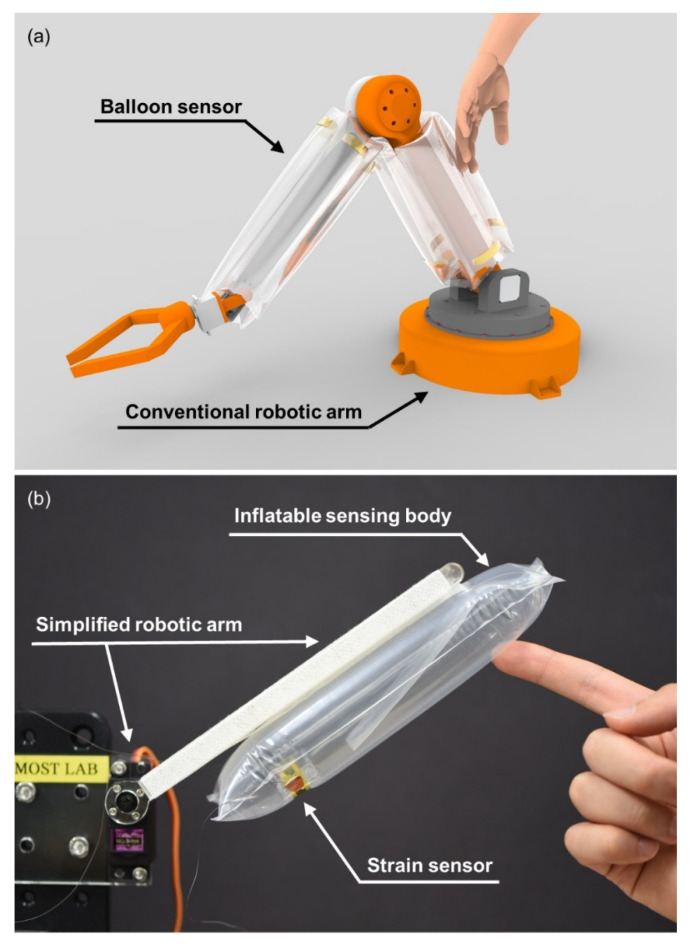
(**a**) Concept design of an application of balloon sensors covering a robotic arm; (**b**) the prototype of the balloon sensor integrated with a simplified robotic arm.

We demonstrate the design of an inflatable body and a crack-based strain sensor, a model of the sensor for comparing the data from experiment and simulation, results verifying its sensing performance and the model, and implementation of the sensor onto a simple robotic arm that can avoid objects by gently touching them. Figure 1 describes the concept design of a conventional robotic arm integrated with balloon sensors and a prototype of a simple robotic arm. The single balloon sensor in the prototype detects external contact as a form of pressure sensor. Arrays of the sensors in concept design are expected to function as tactile sensors providing information for the location of applying force as well.

## 2. Fabrication and Design

### 2.1. Fabrication

**Figure 2:**
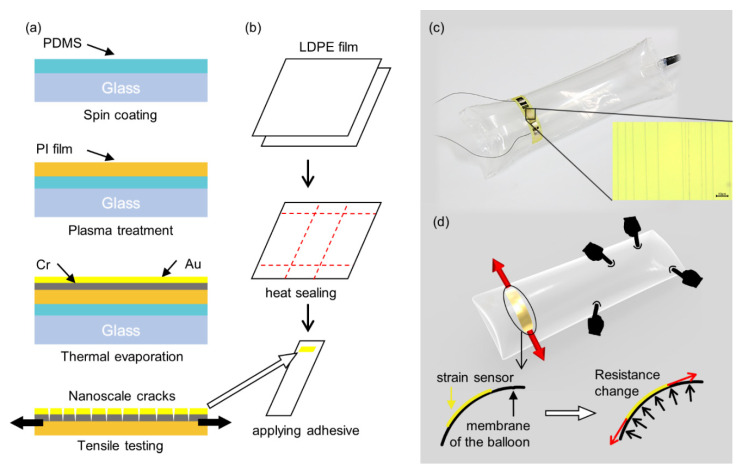
Schematic illustration of a fabrication process for the balloon sensor; (**a**) the Fabrication process for the crack-based strain sensor; (**b**) prepare two sheets of polymer film (LDPE), apply heat by a soldering iron or a heat sealer (200 °C) to the desired parts and cut out the rest. Attach the highly sensitive strain sensor to the inflatable; (**c**) a photograph of the prototype of the fabricated balloon sensor with the microscopic image of the strain sensor; (**d**) schematic design of the sensor illustrating its sensing mechanism.

Figure 2a,b illustrates the fabrication process of the balloon sensor. The process is mainly divided into the fabrication of the inflatable structure and crack-based strain sensor.

First, 7.5-µm thick PI film was attached onto glass coated with polydimethylsiloxane (PDMS), which helps uniform thermal deposition on the PI film. Then, the PI film was plasma treated to improve the bonding between its surface and the metal to be deposited. Second, a layer of Cr was deposited onto the surface of PI, followed by deposition of a layer of Au through thermal evaporation. The thicknesses of deposited Cr and Au layer were 50 nm and 30 nm, respectively. Third, the strain sensor was extended by 2% of its initial length for approximately 100 cycles to generate cracks.

After fabrication of the strain sensor, the inflatable structure on which the strain sensor is to be laminated needs to be fabricated. First, we prepared two sheets of LDPE film or off-the-shelf LDPE air cushion, which can be readily obtained as the body of the sensor. LDPE is a thermal plastic material that can be laminated by a simple heat-sealing process without an adhesive layer. Second, the desired line in the film was heated by a heat sealer and the temperature was set to 200 °C. A piece of LDPE was sealed together as the air inlet. The size of the inflatable was designed to be 20 × 6 cm^2^ and 35 × 10 cm^2^. Third, the remaining part of the film was cut out, and the fabricated strain sensor was laminated onto it by soldering iron or the polymer adhesive. The adhesive was applied only on each end of the strain sensor to avoid any additional layer in the middle between the strain sensor and the balloon. This is because applying the adhesive to the entire area of the strain sensor restricts the strain of the sensor. A copper wire was connected at the end of the strain sensor using a conductive epoxy. Finally, the air at the desired pressure was pumped into the inflatable. The sensing area expands to the entire membrane of the balloon and can be customized by designing the shape of the balloon. In this study, we fabricated a simple cylindrical balloon shape and tested its performance and characteristics.

### 2.2. Sensing Principle

The sensing mechanism of the balloon sensor is shown in Figure 2c,d. When a contact force is applied anywhere on the membrane of the balloon sensor, the pressure inside the balloon increases. Increasing the pressure then expands the balloon. As the balloon expands, tensile force is applied at the ends of the attached crack-based strain sensor to be extended. Crack-based strain sensors have parallel arrays of nanoscale crack that widen by stretching. Widening of cracks results in change in electrical conduction which can be measured as resistance. The strain sensor is an ultra-sensitive strain sensor with a reported gauge factor of up to 100 k [27]. The high sensitivity of the strain sensor allows the use of a stiff membrane of the balloon to increase the payload of the soft inflatable robot. Although the strain is small owing to the stiff membrane of the balloon, the sensitivity of the strain sensor is sufficiently high to detect a small strain. The change in the resistance can be calculated to predict the pressure change inside the inflatable or applied force.

### 2.3. Crack-Based Strain Sensor

**Figure 3:**
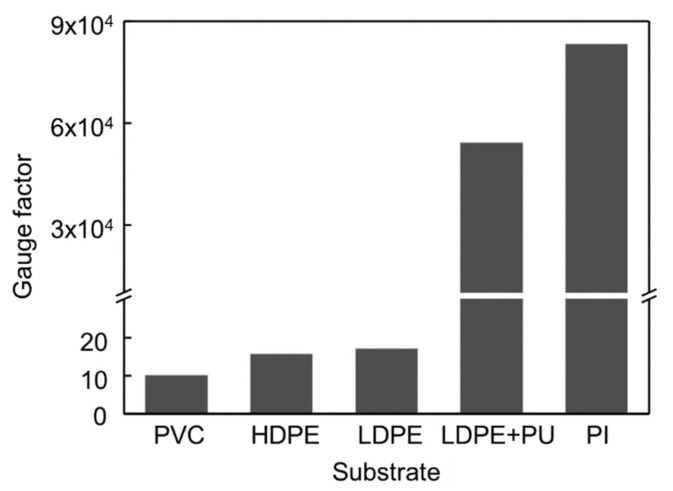
Comparison of gauge factor of crack-based strain sensors with different substrates.

To detect the contact force caused by the change in resistance of the strain sensor, it is important to select a highly sensitive strain sensor whose resistance changes for small deformation of the balloon caused by any small-scale force. Various crack-based strain sensors were fabricated and compared with others. The substrate films used to fabricate strain sensors were polyvinyl chloride (PVC), PET, LDPE, LDPE with spin-coated polyurethane (PU), and PI films. In this study, the highest gauge factor was achieved with the PI film as shown in Figure 3. The gauge factor of the strain sensor used in this study exceeded 80,000 at a strain of 2%. Note that at a strain of 0.2%, the gauge factor was measured to be only 260 which is used in modeling section because of the nonlinearity of the resistance of the strain sensor. The thickness of the strain sensor is also a critical factor for sensitivity [28]. The thinner strain sensors are more advantageous because they can be easily deformed by the stress applied to the sensor. Polyimide (PI) film with a thickness of 7.5 µm was used to fabricate the strain sensor. Its thickness is approximately ten times lower than that of the inflatable body of the LDPE film, which is 80 µm thick. The PI film is also suitable for fabricating the strain sensor because the PI film is less affected by high temperatures than the LDPE film. Thermal evaporation of metals is one of the processes involved in the fabrication of strain sensors. This requires high temperature and affects the LDPE film, while thermal evaporation results in fluctuations in the sensitivity of the sensor.

### 2.4. Customizable Inflatable Body

The inflatable body is fabricated with a relatively stiff and inextensible polymer film, such as polyethylene (PE). A stiff and inextensible film improves robustness and increases the payload of the soft inflatable robot. Soft silicone or urethane rubbers, which are inherently soft and highly extensible, are also suitable materials for balloons. However, large deformations of the materials cause difficulties in modeling and a comparatively low payload. In addition, multiple molding processes are required to create a hollow balloon shape. In this study, LDPE was used as a soft inflatable body. LDPE is commonly used in air pouch, which acts as a filler in packaging to protect the packaged items during transportation. It is easily accessible, cheap, and fabricated by a simple heat-sealing process, as described in the fabrication section.

## 3. Experiments and Results

**Figure 4:**
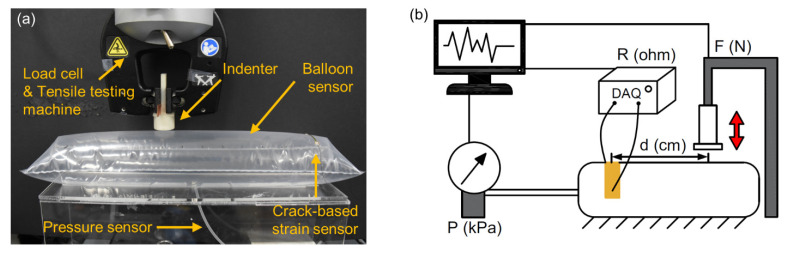
Photograph (**a**) and schematic illustration (**b**) of the experimental setup for sensor characterization.

To measure the response of the balloon sensor, an experimental setup was prepared, as shown in Figure 4. Using a tensile testing machine (3342 UTM, Instron Co., Norwood, MA, USA), the force was applied to the balloon sensor with an indenter. A commercial pressure sensor was connected to the balloon to compare it with the actual pressure inside the balloon. During each test, a data acquisition device (SIRIUS-SYSTEM, DEWESoft Korea, Ltd.), pressure sensor (MPX5100ap), and load cell built in the tensile testing machine measured three types of signals simultaneously: resistance of the balloon sensor, pressure inside the balloon, and compressing force on the balloon, respectively. With this experimental setup, we first tested a balloon sensor with a size of 35 × 10 cm^2^.

**Figure 5:**
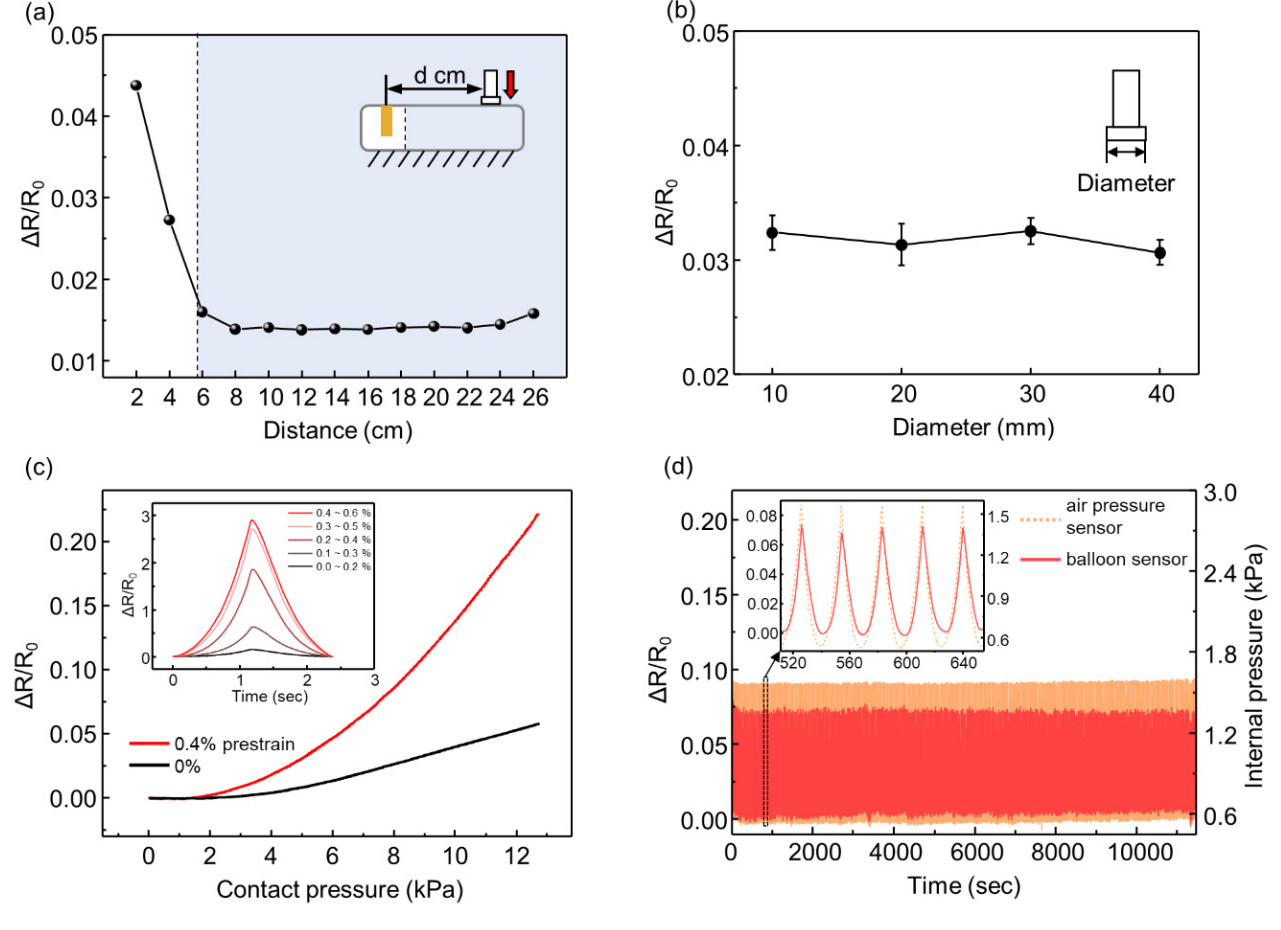
(**a**) Relative changes in the resistance of the balloon sensor for different locations of applied force; (**b**) relative changes in the resistance for different areas of the indenter; (**c**) relative changes in the resistance as a function of contact pressure with a pre-stretched strain sensor and not-stretched sensor; (**d**) response of the strain sensor integrated with the balloon sensor and internal pressure of the balloon for a cyclic test.

After confirming that the location of the contact force does not affect the response in a certain range of the balloon sensor, we pressed the balloon sensor with different areas of the indenter (diameters of 10, 20, 30, and 40 mm) with a contact force of 6 N, as shown in Figure 5b. The results show that with the same magnitude of force, the area of the indenter does not affect the response of the balloon sensor. This suggests that the total deformation of the balloon depends on the magnitude of the contact force, even with different areas of contact force.

To increase the sensitivity of the balloon sensor, we attached the strain sensor such that the strain sensor was pre-stretched (0.4%), as shown in Figure 5c. Owing to the nonlinearity of the crack-based strain sensor, it is advantageous to use the higher strain range of the sensor, as shown in the inset graph in Figure 5c. To attain a higher strain range, we folded the membrane of the balloon before attaching the strain sensor to the pre-strain of the sensor. When the balloon is inflated, the folded membrane of the balloon stretches the strain sensor more than just attaching the sensor to the plain membrane of the balloon. The slope (sensitivity) of the red line (0.4% pre-strain) was steeper than that of the black line (0% pre-strain). Over 0.4% of pre-strain, the strain sensor is unstable to maintain its initial resistance even for constant internal pressure. Furthermore, we demonstrated the high durability of the balloon sensor under loading–unloading cycles, as shown in Figure 5d. The constant change in internal pressure implies that the room temperature (25 °C) was maintained constant enough not to affect the internal pressure. Compared with the internal pressure of the balloon, the resistance amplitude of the balloon sensor also exhibited negligible changes.

### 3.1. Response to Various Forces

**Figure 6:**
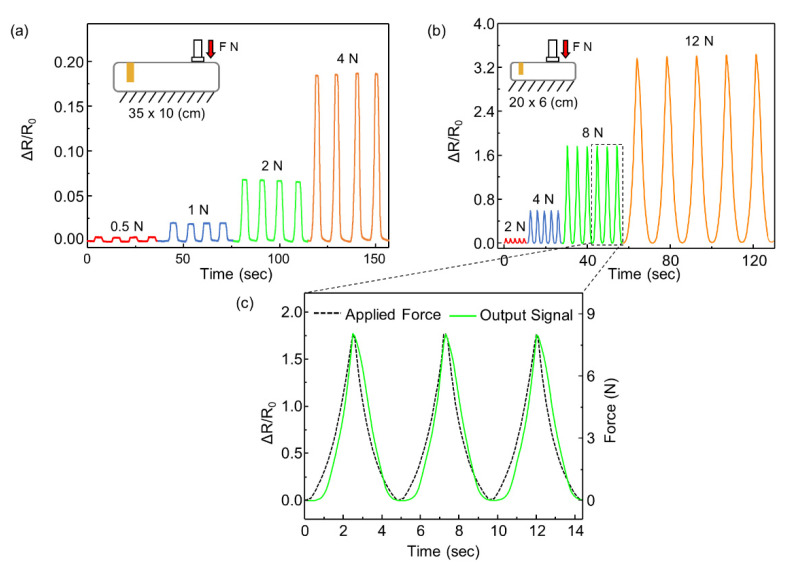
(**a**) Response of the balloon sensor (35 × 10 cm) for the various applied forces of 0.5 N, 1 N 2 N, and 4 N; (**b**) response of the balloon sensor (20 × 6 cm) with 2 kPa of internal pressure for the various applied forces of 2 N, 4 N 8 N, and 12 N; (**c**) enlarged graph for comparison of response of the balloon sensor and load cell.

To verify that the resistance of the balloon sensor is effectively changed by the incremental force, we conducted an experiment in which various forces (0.5, 1, 2, and 4 N) were applied onto a balloon sensor with an initial pressure of 0.5 kPa. The responses were stable, continuous, and noise-free, as shown in Figure 6a. The relative changes in the resistance did not show a linear increase with increasing force. This is because of the nonlinearity of the resistance change of the strain sensor. The applied force and internal pressure showed linearity, which implies that the relationship between these two is not the cause of the nonlinearity. The graph of the resistance of the strain sensor as a function of the strain is exponential.

We also fabricated a smaller balloon sensor (20 × 6 cm^2^) with an internal pressure of 2 kPa. The smaller size of the inflatable body indicates that the resistance change is higher than the external force, as shown in Figure 6b,c. The deformation of the balloon caused by the contact force contributed to the change in the internal pressure of the volume. Under a smaller volume, the pressure increases more with the same volume decrease. A high change in pressure results in a significant change in the resistance of the strain sensor. Furthermore, the balloon sensor with a higher initial pressure sensor (2 kPa) endured a larger payload.

**Figure 7:**
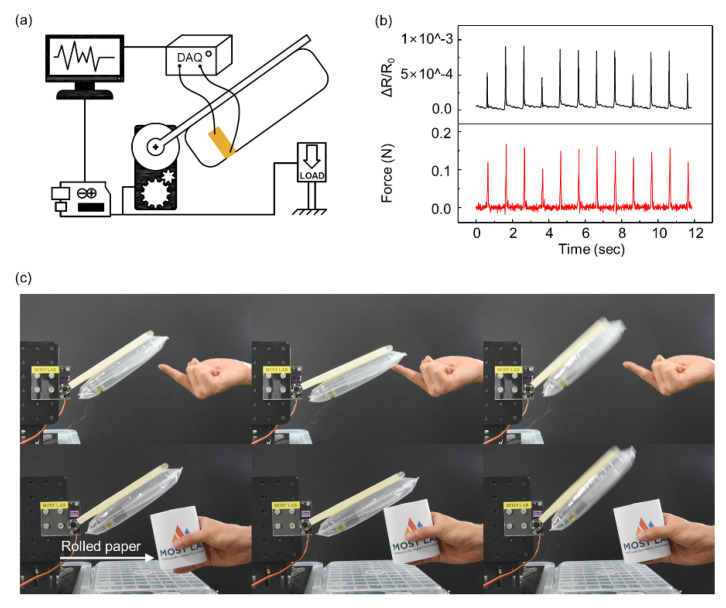
(**a**) Schematic illustration of the experimental setup for the simple robotic arm integrated with the balloon sensor. (**b**) Comparison between the applied force from the load cell (red line) and relative change in resistance (black line). (**c**) A simple robotic arm consisting of the balloon sensor and servo motor detecting gently touching fingers and a sheet of rolled paper. Yellow arrows indicate the movement direction of the balloon senor.

We tested the performance of the balloon sensor with the other experimental setup, as shown in Figure 7a. The balloon sensor was attached to a simple robot arm controlled only by a single servo motor (MG 946R). While measuring the resistance of the balloon sensor by the data acquisition (DAQ) device, the load cell (BCL-1L) measured the load applied by the moving balloon sensor. The microcontroller (Arduino Uno) controlled the servo motor such that the balloon sensor instantly moved backward when the load cell detected any contact force higher than 40 mN. The value of 40 mN is the lowest value of the force that the servo motor can respond to owing to the limitation of the performance of the servo motor and the sensitivity of the load cell. The graph in Figure 7b indicates that as the load cell measured force between a value of 100 mN and 180 mN, the resistance of the balloon sensor was well matched with the data of the force.

By connecting the microcontroller to the balloon sensor with an analog to digital converter (ads1115), the servo motor was directly controlled by the data obtained from the balloon sensor, as shown in Figure 7c. The resolution of the ads1115 is 16 bits. The servo motor rotated in the direction opposite to the moving direction when the resistance of the balloon sensor changes by contacting any object. Using Arduino Uno with the analog to digital converter (ads1115), we can measure force as small as 100 mN without significant noise that affects control of the servo motor. The balloon sensor was able to move backward by detecting a finger and a sheet of rolled paper gently touching it, without being damaged (Video S1). The balloon sensor also showed sensing of any contact force over the entire membrane area.

### 3.2. Modelling

**Figure 8:**
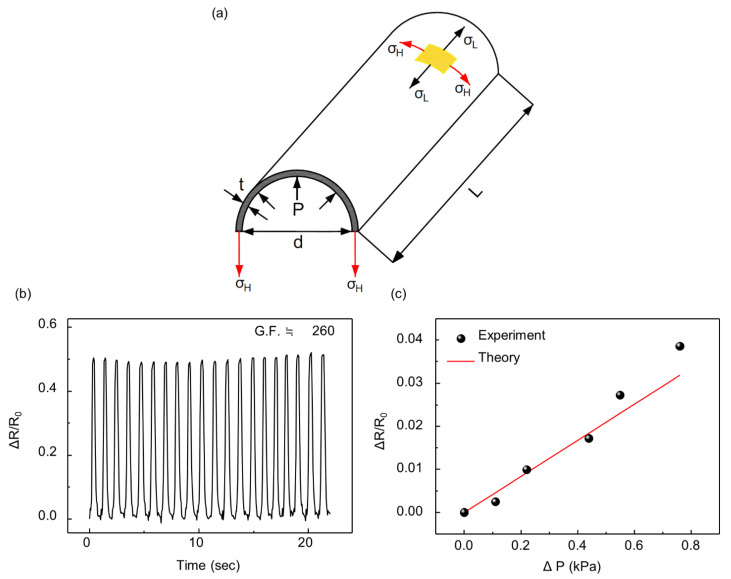
(**a**) Schematic design of the sensor indicating each parameter; (**b**) Relative changes in the resistance of the crack-based strain sensor under a tensile test; (**c**) Comparison between experimental (black dot) and theoretical data (red line).

The shape of an inflatable body determines the mathematical model to predict the relationship between the inside pressure of the balloon and the resistance of the strain sensor. When the inflatable structure composed of two sheets of film is inflated, it becomes a 3D structure with an elliptical cross section. The cross-section of the inflatable body becomes almost a circle on sufficient inflation because of the uniform pressure on the membrane. Therefore, we assumed that if the width of the inflatable is sufficiently short compared to the length, the inflated structure can be considered as a cylinder, as shown in Figure 8a. After this assumption, the stress in the direction of length ($\sigma_{L}$) and circumference ($\sigma_{H}$) is defined as follows:

(1) $$\sigma_{H}=\frac{T}{t}=\frac{PD}{2t}$$

(2) $$\sigma_{L}=\frac{PD}{4t}$$

where *P* is the inside pressure of the inflatable, *D* is the diameter of the cylinder, *t* is the thickness of the film, and *T* is the tensile force per length. The strain of the strain sensor in the circumferential direction is derived from (1) and (2) as follows:

(3) $$\varepsilon_{H}=\frac{1}{E}(\sigma_{H}-\nu \sigma_{L})$$

where $\varepsilon_{H}$ is the strain in the circumferential direction, $E_{eq}$ is the equivalent Young’s modulus of PI and LDPE, and ν is Poisson’s ratio of the film. It is not necessary to derive $\varepsilon_{L}$, which is the strain in the direction of length, because the strain sensor is laminated on the balloon such that the effect of $\varepsilon_{L}$ on resistance change is trivial compared to that of $\varepsilon_{H}$. The nanoscale cracks broadened only when the strain sensor was extended in the direction of $\varepsilon_{L}$. To determine the relationship between the pressure and resistance, it is necessary to define the strain as a function of resistance. The definition of the gauge factor was used to relate the strain and resistance.

(4) $$GF=\frac{\Delta R/R_{s0}}{\Delta l/l}=\frac{\Delta R/R_{s0}}{\varepsilon }$$

Since $\varepsilon =\Delta R/(R_{s0}GF)$ from (4), where *GF* is the gauge factor of the strain sensor, $R_{s0}$ is the initial resistance before the strain, and the relative change in resistance can be derived by substituting strain as follows:

(5) $$\Delta R/R_{s0}=\frac{(GF)PD}{4E_{eq}t}(2-\nu )$$

Equation (5) is derived as the relation between the resistance of the strain sensor and the inside pressure of the balloon. However, rather than using, $R_{s0}$ which is the initial resistance of the strain sensor before the strain, it is more appropriate to use the initial resistance ($R_{0}$) of the strain sensor at the initial pressure ($P_{0}$) inside the inflatable. This is because in every experimental result, the initial resistance is measured at the initial pressure and not before the strain. The strain sensor is already stretched to some degree at the initial state of the balloon. At the initial state of the balloon sensor, $R=R_{0}$ and $P=P_{0}$ in (6).

(6) $$\frac{\Delta R/R_{s0}}{R_{s0}}=\frac{(GF)P_{0}D}{4E_{eq}t}(2-\nu )$$

Because $R_{s0}$ is calculated from (6), $R_{s0}$ can be replaced in (5) as follows:

(7) $$\frac{\Delta R/R_{0}}{R_{0}}=\frac{\left(GF)(P-P_{0})D(2-\nu \right)}{(GF)P_{0}R_{s0}(2-\nu )+4E_{eq}t}$$

where *GF* = 260.04, $E_{eq}$ = 1 GPa, which is the value between those of LDPE and PI, *t* = 0.0875 mm, *D* = 37 mm, ν = 0.4, and $R_{s0}$ = 19.910 Ω. The *GF* was obtained by tensile testing of the strain sensor with a strain of 0.2%, as shown in Figure 8b. The experimental result is compared with the result of the model from (7), as shown in Figure 8c. During the experiment, the balloon was compressed by 1.5 N force at a distance of 6 cm from the strain sensor with an initial pressure of 2 kPa. However, when the balloon was subjected to high external pressure, the model did not match the experimental results. Owing to the nonlinearity of the strain sensor, the resistance increases dramatically at high pressure, which indicates that a new definition of the gauge factor for the crack-based strain sensor is required.

## 4. Conclusions

In this work, we present the design and fabrication of a balloon sensor with an experiment to demonstrate its characteristics. Focusing on the practical use of the sensor, we designed it with a large sensing area compared to the actual sensing part and with light weight, which is approximately 2 g. The simple and cheap fabrication process adds to the practical use of soft robots. The strain sensor used in this work is highly sensitive, flexible, and thin enough to be employed in the balloon sensor. Our balloon sensor shows acceptable responses to various types of force and demonstrates its potential as a soft robot by responding and detecting surrounding objects with only a small contact force. The experiments show that the balloon sensor maintains a stable response with no noticeable delay in sensing. A mathematical model for the balloon sensor was introduced to predict the internal pressure.

However, the balloon sensor faced several challenges. The balloon sensor cannot provide information for the location of the applied force. The sensor in current work is limited to providing a certain sensing area (d = 6 ~ 26 cm). Second, the strain sensor was exposed to external damage. Because the thickness of the metal is less than 100 nm, small damage might be critical. Third, the explained model is limited to small deformations of the balloon sensor. A new equation for explaining the nonlinear relationship between the strain and the resistance of the strain sensor is required.

Future work for the balloon sensor includes developing solutions for the above-mentioned problems. Using more than one strain sensor with additional modelling might enable detection of the location of the force. Encapsulation or lamination of the strain sensor to the inside face of the balloon might be helpful in preventing possible damage to the strain sensor. The sensitivity of the balloon sensor might be increased by changing the geometry of the strain sensor such that more stress is concentrated on the strain sensor. In addition, the design of a more developed actual soft robot controlled based on the balloon sensor will be considered in future work.

## Data Availability

Not applicable.

## Supplementary Materials

The following are available online at https://www.mdpi.com/1424-8220/21/6/2163/s1, Video S1: Balloon sensor integrated with the simplified robotic arm.
